# Preservation of *Lycopersicum esculentum* (Tomatoes) with Extracts of *Annona muricata* (Soursop) and *Hibiscus sabdariffa* (Roselle Plant)

**DOI:** 10.21315/tlsr2022.33.1.1

**Published:** 2022-03-31

**Authors:** Temitope T. Banjo, Omowunmi R. Oluwole, Victoria I. Nzei

**Affiliations:** 1 Department of Biological Sciences, Crawford University, PMB 2001, Igbesa, Ogun State, Nigeria; 2 Department of General Studies, Federal College of Agriculture, PMB 724, Akure, Ondo State, Nigeria; 3 Department of Biological Sciences, Wellspring University, PMB 1230, Irhirhi Road, Edo State, Nigeria

**Keywords:** Preservation, Soursop, Roselle Plant, Tomatoes, Spoilage Microorganisms

## Abstract

Tomatoes are very important vegetable crops in the world but with a perishable nature. Due to its highly perishable nature, various methods have been investigated to increase its shelf life while still preserving its qualities. Therefore, this study investigated the potentials of the parts of *Annona muricata* and the calyces of *Hibiscus sabdariffa* in the preservation of tomato fruits. Tomato fruits were washed and treated with powdered, aqueous and ethanolic extracts of the leaves, seeds, bark of the *Annona muricata* and calyces of *Hibiscus sabdariffa* at different concentrations of 3%, 5%, 6%, 9% and 12% w/v. The tomato fruits were placed in well-aerated baskets for a period of 35 days during which organoleptic and microbial analysis were carried out. The different treatments with *Annona muricata* and *Hibiscus sabdariffa* had significant effects on the preservation of the tomato fruits at *p* < 0.05. The leaves of *Annona muricata* proved most effective preserving up to 50% of the tomatoes after the monitoring period. Moreover, 6% (w/v) of the aqueous extract of the leaves of *Annona muricata* resulted in a preservation rate of 75% of the tomato fruits. The spoilage microorganisms isolated from the tomato fruits are *Candida krusei, Candida sp., Bacillus subtilis and Bacillus* sp. The results of this research carried out shows that the extracts of *Annona muricata* and *Hibiscus sabdariffa* had significant preservative activities on the tomato fruits (*p* < 0.05), thus minimising wastes and economic loss to the farmers and country in general.

**Highlight**

- The powdered leaf of *A. muricata* preserved up to 50% of the tomatoes 35 days after treatment.
- Six percent (6%) Aqueous extracts of *A. muricata* leaves preserved up to 75% of the tomatoes 15 days after treatment.
- The spoilage microorganisms isolated from the tomato fruits are *Candida krusei*, *Candida* sp., *Bacillus subtilis* and *Bacillus* sp.

## INTRODUCTION

Tomato is a nutritious fruit of the plant *Solanum lycopersicum* which belongs to the family *Solanaceae* (Maseret *et al.* 2012). This vegetable is cultivated in Nigeria and in high demand as a component of salad and sauce/soup by the different tribes of the country (Ploey & Heuvelink 2005). There are different varieties of tomato fruits with different colours which may be yellow, orange-green or purple (Ebimieowei *et al*. 2013). The fruit contains high amount of carbohydrates, fats, organic acids, water, minerals, vitamins and pigments. It is estimated that ripe tomato fruits contain approximately 94% water, 4.3% carbohydrates, 1% protein, 0.1% fat, 0.6% fibre and vitamins (Wogu & Ofuase 2014). The nutrients support the growth of microorganisms such as fungi and bacteria, which produce enzymes that degrade the nutrients (Maddox 1998). Tomato fruits contain a lot of water which makes them more susceptible to spoilage by microorganisms. Also, the high-water content makes storage and transportation of this vegetable difficult. The microorganisms reduce not only the nutritional value but also the market value of tomato fruits (Wogu & Ofuase 2014). Tomato fruits are of a highly perishable nature, with a short shelf life of between 12 h to 72 h (Ejale & Abdullah 2004). In most developing countries, microbial infestation of tomatoes can occur during the harvesting period, post harvesting, handling, storage, transportation and processing by customers (Yeboah 2011). Contamination of tomato fruits also occur by exposing them on benches and baskets in the open markets for customers (Baiyewu *et al*. 2007). Some studies have been carried out on bacteria associated with tomatoes and tomato products in some countries. A study carried out by Ajayi (2013) in the United States has revealed that *Clostridium* sp., *Staphylococcus* sp. and *Bacillus* sp. were predominant bacteria isolated from both canned and raw tomatoes. In India, a study carried out on tomato puree revealed the presence of *Klebsiella* sp., *Proteus mirabilis*, *Vibrio* sp. and *Pseudomonas* sp. (Garg *et al*. 2013). In Nigeria, Wogu and Ofuase (2014) isolated *Bacillus subtilis*, *Klebsiella aerogenes*, *Pseudomonas aeruginosa*, *Salmonella typhi*, *Proteus mirabilis* and *Staphylococcus aureus* from spoilt tomatoes in Benin City. A similar study also revealed high levels of *Staphylococcus* sp. (22.5%), *Bacillus* sp. (20%) and *Escherichia coli* (15%) in Lagos State, Nigeria (Ogundipe *et al*. 2012). Due to the high perishable nature of the fruits, a lot of them rot before they get to the various areas of the country where they are not cultivated and where the demand is high (Irokanulo *et al*. 2015). Different methods of preservation have been utilised over the years to curb these economic losses resulting from the perennial spoilage of tomato fruits. However, most developing countries like Nigeria cannot afford the use of cold storage facilities for the preservation of this nutritious and important fruit, which may be due to lack of capital lack of technical knowledge or epileptic power supply (Thirupathi *et al*. 2006). Another challenge is limited finances to invest in such storage facilities (Sood *et al*. 2011). The awareness of the effects of chemical preservatives like Sodium hypochloride, Sodium metabisulphite and Calcium chloride on the health of consumers has resulted in an intensified effort on the use of plant extracts in the preservation of tomato fruits (Irokanulo *et al*. 2015). However, little attempt has been made in the use of plants, which are multipurpose, cheap, easy to use and have found tremendous use in food and medicine to preserve the fruit (Hosea *et al*. 2017).

The use of plant extracts in food preservation offers some benefits when compared to other methods of preservation which includes: they are readily available, they are cheap, they improve the economic value of the food products and they are tolerable than the chemical preservatives (Bautista-Banos *et al*., 2002). The powder of Neem (*Azadirachta indica*) leaves has been reported to be effective against spoilage microorganisms thus extending the shelf life and quality of tomato fruits in storage (Hosea *et al*. 2017). Furthermore, Ijato *et al.* (2011) reported the antimicrobial effect of *Vernonia amygdalina* and *Tridax procumbens* in in-vitro control of tomato (*Lycopersicum esculentum*) post-harvest fruit rot. These plant extracts prevent biodeterioration of the tomato fruits by microbial agents of deterioration such as fungi, bacteria and viruses which ultimately helps to avert losses of farm produce and income to the farmers and the country at large (Ejale & Abdullah 2004). However, information on the use of soursop and Roselle plant is scanty.

The soursop plant (*Annona muricata*) is a well-known tropical plant whose parts have been utilised as a component of some herbal concoctions (Raybaudi *et al*. 2015; Rosemary *et al*. 2017). The active agent responsible for the antimicrobial property of soursop seeds and leaves has been identified as actinogens (Viera *et al*. 2010). Soursop leaves contain flavonoids, tannins, alkaloids, saponins, calcium, phosphorus, carbohydrates, vitamins A, B, and C, phytosterol and calcium oxalate (Edeoga *et al*. 2005; Abdul-Wahab *et al*. 2018). Many active chemical compounds, especially terpenoids, are thought to have potential as an antibacterial, antidiabetic potentials, antihypertensive properties, antioxidative and anticancer effects (Gavamukulya *et al*. 2014; Adefegha *et al*. 2015; Sari *et al*. 2018). The leaves are also used to treat several types of bacterial disease, such as pneumonia, diarrhea, urinary tract infection, and various skin diseases (Gajalakshmi *et al*. 2012). Ethanol extract of soursop leaf (*A. muricata* L.) had antibacterial activity against ATCC® 35668™ *Streptococcus mutans* with minimum inhibitory concentration (MIC) at the concentrations of 125 mg/mL (Rahman *et al*. 2017). Soursop leaf ethanolic extract has shown the highest antibacterial activity toward *Pseudomonas aeruginosa* and *Staphylococcus aureus* (Vijayameena *et al*. 2013). The methanolic and aqueous extract of the leaves of this plant *Annona muricata* possess antimicrobial activities against some bacteria among which are *Staphylococcus aureus*, *Escherichia coli* and *Klebsiella pneumonia* (Panthak *et al*. 2010).

The Roselle plant (*Hibiscus sabdariffa*) is a shrub with the characteristic five petals, funnel shaped flower belonging to the malvaceae family (Brunold *et al*. 2004). The different parts of this plant have many applications. The Roselle plant has been used for therapeutic purpose over the years (Okereke *et al*. 2015). In addition, the seeds of Roselle plant are very nutritious food source because it is rich in protein, calories, fiber and micronutrients (Al-Wandawi *et al*. 1984; Balami 1998). Roselle is also rich in organic acids, minerals, amino acids, carotene, vitamin C and total sugar in its calyx, leaves and seeds at variable levels depending on the variety and geographical area (Mady *et al*. 2009). According to Mishra *et al.* (1999), a number of compounds have also been isolated and characterised from Roselle including flavonoids, anthocyanidins, triterpernoids, steroids and alkaloids. The antibacterial potential of hibiscus on *Campylobacter* species has also been reported (Yin & Chao 2008). Furthermore, the ethanol extract of the dried leaves of Roselle showed an *in vitro* inhibitory effect against several bacterial strains, such as *Staphylococcus aureus*, *Bacillus stearothermophilus*, *Micrococcus luteus*, *Serratia mascences*, *Clostridium sporogenes*, *Escherichia coli*, *K. pneumonia*, *Bacillus cereus* and *Pseudomonas fluorescence* (Olaleye 2007). In agriculture, the leaves of this plant are used as fodder and fibre in feeding animals (Wong *et al*. 2002); the seed can be used to feed the poultry as well as sheep and the residue from the seeds oil extracted can also be used to feed cattle and chicks (Babalola *et al*. 2001). Besides its extended consumption as a beverage and its uses in the food industry, Roselle is also used in animal feed, nutraceuticals, cosmetics and pharmaceuticals (Wogu & Ofuase 2014; Borras *et al*. 2015).

Biodeterioration of tomato fruit results in heavy quantitative and nutritional losses to farmers and consumers as well as the rural and urban dwellers far from areas of production that will have to pay more for few healthy fruits that gets to them (Ejale & Abdullah 2004). Therefore, its preservation and storage is important to the economy of individual homes, farmers and the country considering the vital role it plays in the health of people (Irokanulo *et al*. 2015). This study seeks to develop a more health-friendly alternative method of preservation in which tomato fruits can be properly preserved to avoid wastage while still preserving its taste and nutritional value. Hence, this study investigates the potentials of Roselle and soursop plants in the preservation of tomato which is a nutritious fruit with several health benefits.

## MATERIALS AND METHODS

### Plant Materials

Dry calyces of the Roselle plant (*H. sabdariffa*) were obtained from Ogba market in Benin City. The soursop (*A. muricata*) and tomato (*Lycopersicum esculentum var brandywine*) fruits were obtained from Oba market, Benin City. Matured leaves and barks of the soursop plant were obtained from Wellspring University, Benin City, Edo State, Nigeria.

### Collection and Preparation of Plant Materials

The leaf and bark of the soursop plant were air dried for two weeks at room temperature. The seeds were removed from the fruits, washed with water were also air dried for two weeks. The dried plant materials were milled into powdery form using a waring blender. This was sieved through 1.0 mm sieve to obtain a fine powder which was kept in an airtight container until further use. The calyces of the Roselle plants were not blended but stored in a dark cupboard.

The tomatoes selected were fully ripe and red in colour. Those with deformity, pigmentation, wrinkle (with a thumb slide), darkened or with bruised areas on or under the skin of the tomatoes were rejected.

### Preparation of Extracts

#### Preparation of ethanolic extracts

The test solution of each extract was prepared by dissolving the 10 g of the plant extracts in 100 mL (10% w/v) ethanol inside a conical flask and corked tightly (Kator *et al*. 2019). Each of the conical flasks was labeled as EEL (ethanolic extract of leaves), EES (ethanolic extract of seeds) and EEB (ethanolic extract of bark). The conical flasks were placed on a vibrator for 48 h to allow for proper extraction of the plant materials.

#### Preparation of aqueous extracts

To prepare the aqueous extracts, 10 g of plant materials was measured into 100 mL (10% w/v) of previously sterilised distilled water (Kator *et al*. 2019). The conical flasks were labelled as EWL (water extract of leaves), EWS (water extract of seeds) and EWB (water extract of bark). For the Roselle calyces, 10 g was measured into 100 mL (10% w/v) of distilled water and was labeled as EWZ (water extract of Roselle leaves). The samples were then placed on the vibrator for 48 h.

### Experimental Design

#### Effect of solvent type

This is to determine the influence of extraction solvents on the antimicrobial properties of the leaves, bark and seeds of *A. muricata* and the leaves of *H. Sabdariffa* leaves.

#### Ethanolic extracts of the leaves, bark and seeds of Annona muricata

The tomatoes were washed thoroughly and eight each was placed in three beakers already labelled. The extracts were then poured on the tomatoes in each beaker and the tomatoes were allowed to soak in them for 30 min. After 30 min, the tomatoes were removed from the extracts and placed in well aerated baskets already designated as EEL, EEB and EES.

##### Aqueous extracts of Annona muricata and Hibiscus sabdariffa

The tomatoes were washed thoroughly and eight each was placed in three beakers already labeled. The extracts were then poured on the tomatoes in each beaker and the tomatoes were allowed to soak in them for 30 minutes. After 30 min the tomatoes were removed from the extracts and placed in well aerated baskets already designated as EWS, EWB, EWL and EWZ.

##### Effect of plant powder on tomato preservation

Another set of tomatoes, eight each were also placed in three separate basket and treated with the powder of the sour sop leaves, bark and seeds by coating each of the tomato seeds with the powder. They were labelled as PS (powder of seeds), PB (powder of bark) and PL (powder of leaves), respectively and kept with the others in well-aerated place (Maseret *et al*. 2012). The plant part with optimum preservative ability was selected for further studies.

#### Control

The tomatoes were washed thoroughly and eight was placed in a basket already labelled control (C) without any treatment.

### Effects of Different Concentrations of Aqueous Extracts of *Annona muricata* on the Preservation of Tomato Fruit

The effects of different concentrations of the aqueous extraction of the leaves in the range 3%–12% (3%, 5%, 6%, 9% and 12%) on tomato fruits was carried out. This was monitored for two weeks.

### Isolation and Identification of Spoilage Microorganisms of Tomato

#### Isolation of spoilage microorganisms

Spoilage microorganisms were isolated from the tomato fruits after three weeks of monitoring. Sterile syringe was used to collect the tomato fluid from fruits showing signs of spoilage (Control, EWS and EEB).

##### Preparation of media

Culture media used for this evaluation were nutrient agar and potato dextrose agar for bacteria and fungi, respectively. The media were prepared according to manufacturer’s instructions. The powered nutrient agar of 28 g was dissolved in 1 L of deionised water, allowed to soak for 10 min and then sterilised by autoclaving for 15 min at 121°C. Potato dextrose agar of 39 g was also dissolved in 1 L of distilled water and boiled to dissolve the medium completely before sterilising with autoclave at 121°C for 15 min. The pH of the sample was adjusted to 3.5, after adding 10 mL of lactic acid. The medium was thereafter cooled to 55°C.

##### Isolation of bacteria and fungi

Using standard microbiological technique (serial dilution), 1 mL of the tomato extract was pipetted and mixed in another 9 mL of sterile distilled water in a test-tube. The test-tube was shaken vigorously to homogenize. The exponential dilution continued to the fourth factor (10^−4^). One millilitre (1 mL) of the fourth factor was aseptically transferred and plated in duplicate sets using sterile molten lukewarm nutrient agar. The poured plates were allowed to set and were incubated (Gallenkamp, England) at 37°C (24 h) and 28°C (48 h) for bacteria and fungi, respectively. Sub culturing of distinct colonies were carried out to obtain pure cultures for further identification.

#### Identification of spoilage microorganisms

##### Identification of bacteria

Bacteria isolates from the spoilt tomato fruits were identified according to the methods of Cowan and Steel (1993) for bacteria identification.

1. Colonial morphology: Each bacteria isolate was examined for their colonial appearance and the colonial characteristic were identified by their size, shape, consistency, colour, elevation and opacity.
2. Cellular morphology: Each pure bacteria isolates was stained by Gram staining techniques.

A smear of the isolates was made on glass slide; it was then stained with Crystal violet for 60 s. After the 60 s, the slide was rinsed with water and then stained with Lugol’s iodine for 60 s. The slide was then rinsed with water after 60 s and decolourised with acetone. The slide was stained with safranin for 30 s, rinsed with water and allowed to dry. The slide was then examined under the microscope with oil immersion using ×100 objective lens. The shape, colour and arrangement of the bacteria cell was examined and recorded.

##### Biochemical identification

The following biochemical tests were performed to further characterise the bacteria isolates according to World Health Organisation (WHO 1983).

1. Catalase test: This test was done according to Monica Chessbrough (1994). Loopful of pure inoculums was dipped into 3% hydrogen peroxide; bubble production indicates positive test while no bubble indicates negative result.
2. Sugar fermentation test: Sugar containing medium was inoculated with the pure test isolates and incubated at 37°C for 18h to 24 h. The production of acid and gas as a result of fermentation was shown by changes in the colouration of the medium and gas production with the following sugar solution containing 1% Andrade indicator; glucose, lactose, maltose, mannitol, sucrose and xylose.
3. Oxidase test: Production of cytochrome oxidase by certain bacteria that can catalyse the transport of electron between electron donor-bacteria and redox dye tetramethyl paraphenylene diamine, reducing it to deep purple colouration was done as follows. A piece of filter paper was soaked with prepared oxidase reagent 1% freshly prepared tetramethyl-p-phenylene diamine dihydrochlomide) and a pure colony of the bacteria was smeared on it. Purple colouration of the colony indicates positive result within few seconds; no colour changes indicate negative reaction.
4. Urease test: Ability of different isolates to break urea by production of urease enzyme. Pure colony of the organism was inoculated into urea medium and incubated for 24 h at 37°C to observe red pink colour changes which indicate a positive test.
5. Indole test: This test was done to demonstrate ability of the isolates to decompose amino acid tryptophan to indole. Presence of indole was tested for its reaction with p-dimethyl amino benzaldehyde.
6. Citrate utilisation test: This test was done to demonstrate the ability of the organism to utilise citrate as its only source of carbon. Pure colony of the isolate was inoculated into Simmon-citrate agar medium which contain sodium citrate, an ammonium salt and an indicator bromothymol blue, then incubated at 37°C for 24 h. Blue colouration indicates positive test while original green indicates negative test.
7. Methyl red test: This test was performed to detect the production of acid from glucose which lowers the pH of the medium, resulting in colour changes. A few drops of methyl red were added to overnight glucose phosphate broth with the resultant red colouration indicating a positive reaction.

##### Identification of yeast

The yeast cells isolated from the spoilt tomato fruits were identified according to the method described by Cowan and Steel (1993) and Chessbrough (1991).

### Germ Tube Test

A total of 0.5 mL of serum was placed into sterile test tube. A loopful of the yeast was then inoculated into the serum and incubated at 37°C for 4 h. After incubation, a loopful was placed on glass slide and cover with cover slip and examined under ×40 objective lens for germ tube production.

### Urea Test

A total of 0.5 mL of the overnight broth of the yeast sample incubated at 37°C overnight was added to 5 mL of urea solution containing 1% phenol red as indicator. When colour changes to pink, it indicates positive urea utilisation while yellow colour indicates negative reaction.

### Cycloheximide

A total of 0.5 mL of yeast sample incubated at 37°C over night was added to 5 mL of cycloheximide solution. A loopful was examined under microscope for presence of yeast. Presence of yeast indicates no inhibition by cycloheximide while absence indicates inhibition reaction.

### Glucose

A total of 0.5 mL of yeast sample was added to 5 mL of glucose solution containing 1% phenol red indicator and incubated overnight. A change in colour from pink to yellow indicates positive reaction while pink colour indicates negative reaction.

### Data Analysis

Mean and standard deviation of the duplicated data (N = 2) were analysed while the significance of the effects of the powder, aqueous and ethanolic extracts of the leaf, bark and seed of *Annona muricata* on tomato preservation were determined by using a one-way ANOVA at 95% confidence interval (0.05 level of significance). Significance of the aqueous extract of the calyces of *H. sabdariffa* on tomato preservation was also determined taking *p* < 0.05.

## RESULTS

The results of the preservation potential of *A. muricata* and *H. sabdariffa* plant extracts on tomato fruit in this study were based entirely on the organoleptic tests of the tomato which includes the visual observation, touch and smell. The tomatoes were considered spoilt if there was evidence of softening, wrinkle, tear or microbial growth.

### The Effects of *A. muricata* Powder on Preservation of Tomato Fruits

The tomatoes coated with the bark powder showed 100% spoilage by the 30th day with 3 out of 8 (37.5%) not spoilt by the 25th day. The tomatoes coated with the seed powder showed 100% spoilage by the 35th day with 3 out of the 8 (37.5%) not spoilt by the 30th day. The tomatoes coated with the leaf showed a better and higher degree of preservation with 4 out of the 8 (50%) not spoilt by the 35th day. The control was not treated with any powder and recorded 100% spoilage by the 20th day with only 1 out of 8 (12.5%) still preserved by the 15th day. The powder of the different parts of *A. muricata* showed a significant on the preservation of the tomato fruits at *p* < 0.05. The leaf powder showed greater ability to preserve the tomatoes from spoilage with more than 60% still preserved 25 days post treatment (Table 1). The mean preservation rate of the powder from the leaf, bark, seed and control for the tomatoes for the period of the study was 73.21%, 46.43%, 57.14% and 21.43%, respectively (Table 1).

### The Effects of Ethanolic Extract of *A. muricata* on Preservation of Tomato Fruits

Tomatoes treated with EEL showed 100% spoilage by the 35th day with 2 out of 8 (25%) still preserved on the 30th day. Tomatoes treated with EEB showed better signs of preservation with 2 out of 8 (25%) still preserved by the 35th day. Tomatoes treated with EES also showed good signs of preservation with 2 out of 8 (25%) still preserved by the 35th day. The control was not treated with any powder and recorded 100% spoilage by the 20th day with only 1 out of 8 (12.5%) still preserve by the 15th day. The ethanolic extracts of the seeds and bark of *A. muricata* had a significant effect on the preservation of tomato fruits from microbial spoilage (*p* < 0.05). The seed and bark ethanolic extracts showed greater ability to preserve the tomatoes from spoilage with about 50% still preserved 25 days post treatment (Table 2). The mean preservation rate of the ethanolic extracts of leaf, bark, seeds and control for the tomatoes for the period of the study was 46.43%, 57.14%, 53.57% and 21.43%, respectively (Table 2).

### The Effects of Aqueous Extract of *A. muricata* and *H. sabdariffa* on the Preservation of Tomato Fruits

The aqueous extracts of *A. muricata* and *H. sabdariffa* had significant effects on the extension of the shelf life of the tomato fruits (*p* < 0.05). Tomatoes treated with EWL showed good signs of preservation with 3 out of 8 (37.5%) still preserved after 35 days. The tomatoes treated with EWB also showed good signs of preservation with 2 out of 8 (25%) still preserved after 35 days. The tomatoes treated with EWS also showed good signs of preservation with 2 out of 8 (25%) still preserved after 35 days. The tomatoes treated with EWZ showed better signs of preservation with 4 out of 8 (50%) still preserved after 35 days. Overall, EWZ showed greater ability to preserve the tomatoes from spoilage with 50% still preserved 30 days post treatment (Table 3). The mean preservation rate of the aqueous extracts from the leaf, bark, seed, Roselle plant and control for the period of the study was 64.30%, 62.5%, 62.5%, 71.43% and 21.43%, respectively (Table 3).

### Organoleptic Result

**Figure 1:**
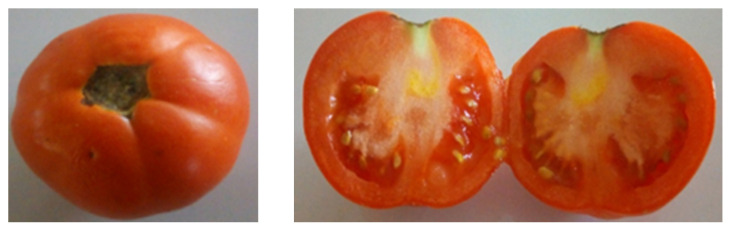
The whole and cut section of tomato fruits after 35 days of treatment.

The preserved tomatoes were washed and examined for organoleptic properties at the end of the experiment (35 days). The tomatoes remained firm to touch, bright, maintaining the usual red colouration (Fig. 1).

### The Effects of Different Concentrations of the Aqueous Extracts of *A. muricata* Leaves on Preservation of Tomato Fruits

The different concentrations of the aqueous extracts of *Annona muricata* leaves exhibited significant effects on the preservation of the tomato fruits (*p* < 0.05). The tomatoes treated with the concentration of 3% showed preservation with 2 out of 4 (50%) still preserved after 15 days. The tomatoes treated with the concentration of 5 g showed 100% spoilage by the 15th day with 1 out of 4 (25%) still preserved after 12 days. The tomatoes treated with the concentration of 6% showed the highest rate of preservation with 3 out of 4 (75%) still preserved after 15 days. The tomatoes treated with the concentration of 9% showed preservation with 1 out of 4 (100%) still preserved after 15 days. The tomatoes treated with the concentration of 12% showed preservation with 2 out of 4 (50%) still preserved after 15 days. Overall, the concentration of 6% (w/v) showed greater ability to preserve the tomatoes from spoilage with 75% still preserved after 15 days post treatment (Table 4). The mean preservation rate of 3%, 5%, 6%, 9% and 12% aqueous extract of the *Annona muricata* leaves on the tomato fruits for the period of the study was 83.33%, 50%, 91.70%, 62.5% and 75%, respectively (Table 4).

### Comparative Studies of the Mean Preservation Rates of Tomato Fruits with Different Treatments of *A. muricata*

There is significant difference in the effects of the powder, ethanolic and aqueous extracts of the different parts of the *A. muricata* on the preservation of tomato fruits (*p* < 0.05). The powder of the leaf of *A. muricata* had the highest mean preservation rate of 73.21. In addition, the aqueous extract of the leaf resulted in a mean preservation rate of 64.3, while the ethanolic extract of the bark resulted in a mean preservation rate of 57.14 (Table 5).

### Identification of Spoilage Microorganisms from Tomato Fruits

**Figure 2:**
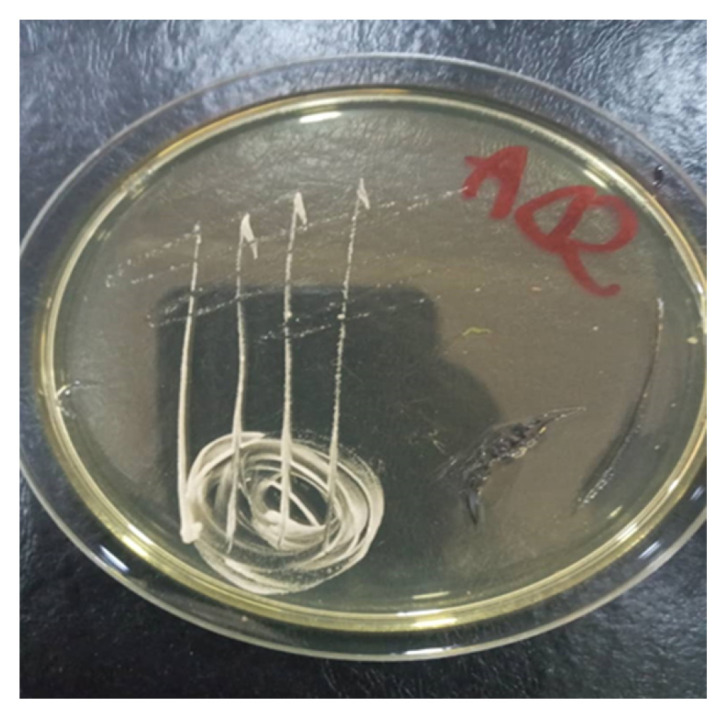
Diagram showing colonial characteristics of *Candida krusei.*

**Figure 3:**
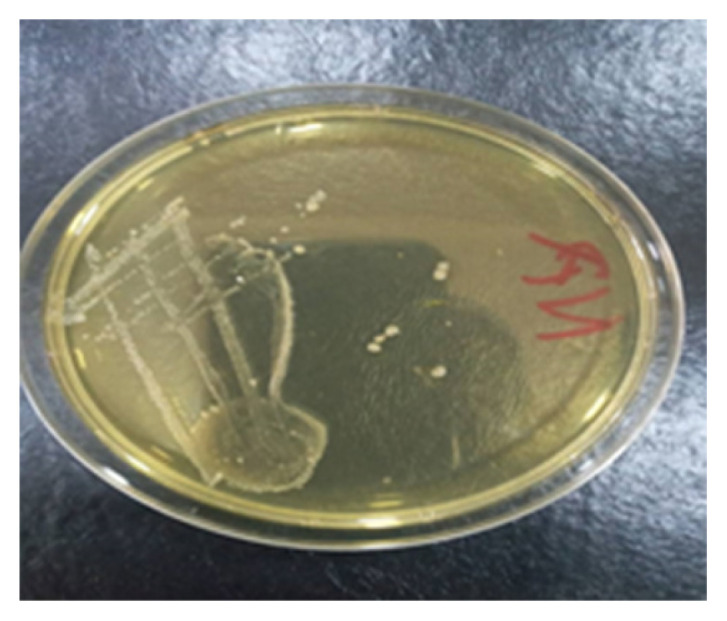
Diagram showing colonial characteristics of *Bacillus subtilis.*

The microorganisms isolated from the spoilt tomato fruits were identified as *Candida krusei*, *Candida* sp., *Bacillus subtilis* and *Bacillus* sp. (Figs. 2 and 3; Tables 6 and 7).

## DISCUSSION

The tomato fruit is a highly perishable food widely used as vegetable. The highly perishable nature of tomato is a problem not just in Nigeria but all over the world. It is therefore important to preserve them in order to increase the shelf life and also make them available all year round.

The phytochemicals present in soursop leaves is predominantly responsible for their antimicrobial effect on spoilage microorganisms. Soursop leaf extract is rich in bioactive compounds such as alkaloids, flavonoids, tannins, steroids and saponins that are antimicrobial in action (Pai *et al.* 2016).

The powder of leaves of *A. muricata* showed the highest rate of preservation after the 35 days of treatment. This is in agreement with the work of Irokanulo *et al.* (2015) who reported the use of leaves, seed and bark of *Moringa oleifera* to preserve fresh tomatoes. The result in this study is similar to the findings of Ejale and Abdullah (2004), who reported that treating tomato fruits with Neem significantly increased their shelf life. This present study shows that the powder of the leaves proved most effective in the preservation of the tomato fruits and that of the bark was the least effective. The leaves possess more phytochemical components than the stem or fruits (Edeoga *et al.* 2005). The leaves of *A. muricata* can therefore be said to be highly antimicrobial compared to other parts of the plant. The ability of soursop leaf powder to minimise the decay of tomato fruits in this study can be attributed to the fact that the soursop powder suppressed the activity of certain fungi that cause spoilage of tomato fruits. This statement is in agreement with Singh *et al.* (1999) who conducted an experiment on the efficiency of crude plant extracts as an alternative to commercial fungicides in the preservation of plant products. This observation is in agreement with the reports of Raheja and Thakore (2002) who reported that extract from medicinal plants like *Allum sativum* (cloves), *Azadirachta indica* (leaves), *Mentha arvensis* (leaves) and *Psoralea Corylifolia* were found most effective in preserving plant fruits from attack by pathogenic and environmental factors. Phytochemical analysis of soursop leaves revealed the presence of acetogenins which is antimicrobial in action due to the effects of their cytotoxic action against the microorganisms (Zafra-Polo *et al.* 1998). These bioactive compounds are responsible for the preservation of the tomato fruits by extending their shelf life since they are anti-microbial in action (Pomper *et al*. 2009).

The ethanolic extracts of the *A. muricata* leaves, seeds and bark showed mean preservation rate of 46.43%, 53.57% and 46.43%, respectively, 35 days after treatment. The ethanolic extracts of the seeds showed greater ability to preserve the tomatoes with 2 out of 8 (25%) still preserved 35 days after treatment. This is in line with the work of Raybaudi *et al.* (2015) who tested the aqueous and alcohol extract of the seeds of *A. muricata* against various microbial strains like *Salmonella enterica* and *Staphylococcus aureus* and some human tumour cell lines. It was also shown that the seeds of *A. muricata* contain several antioxidant compounds such as phenol, morin, flavonoids, ascorbic acids, quercetin, carotenoids and acetogenins which have shown antibacterial activity against several pathogenic and spoilage microorganisms (Vijayameena *et al*. 2013). This antimicrobial activity of the flavonoids against bacteria is accomplished by damaging the cell walls of bacteria consisting of lipids and amino acids. Therefore, the bacterial cell nucleus will undergo lysis and eventual death of the cell (Cushnie & Lamb 2005). Furthermore, the phenolic compounds like tannins inactivate the function of the genetic material and shrinkage of the cell wall leading to the death of spoilage microorganisms (Jannah *et al.* 2017).

The aqueous extracts of the leaves, seeds and bark of *A. muricata* and the calyces of *H. sabdariffa* showed a mean preservation of 64.30%, 62.50%, 62.50% and 71.43%, respectively, 35 days post treatment. This agrees with the work of Rosemary *et al.* (2017) who reported that the leaves of *A. muricata* are capable of inhibiting the growth of several bacteria like *Bacillus subtilis, Candida albicans, Klebsiella pneumonae*; *Aspergillus flavus* and *Fusarium oxysporium.* Furthermore, this observation is in agreement with the antibacterial analysis carried out by Panthak *et al*. (2010) which revealed that the aqueous extracts of the leaves of *Annona muricata* tested against various bacterial strains such as *Bacillus subtilis* ATCC12432, *Staphylococcus aureus* ATCC29213, *Escherichia coli* ATCC8739, *Klebsiella pneumonia* No. 2719, *Staphylococcus pyogenes* ATCC8668 and *Enterobacter aerogenes* NCIM No. 2340 showed positive activities against tested organisms. In a similar study on the use of aqueous extract of Moringa leaves on postharvest shelf life and quality of tomato fruits, Kator *et al*. (2019) reported the ability of the aqueous extract to preserve tomato fruits for 25 days whereas the present study extended the shelf life of tomato fruits to 35 days. Furthermore, aqueous extracts of the selected plant species have been reported to extend the shelf life and enhance the market value of tomato fruits (Balami 1998; Bautista-Banos *et al*. 2002). In addition, Hosea *et al*. (2017) also reported that the calyces of *H. sabdariffa* contains several phytochemicals which is capable of inhibiting the growth of microorganisms and also is highly acidic which prevents many microorganisms from thriving as most do not survive highly acidic conditions.

Overall, aqueous extract of *H. sabdariffa* showed greater ability to preserve the tomatoes than the aqueous extract of *A. muricata*. This may be due to the highly acidic pH of the *H. sabdariffa* extract which was 1.0 when tested. This acidic nature of the extract is able to inhibit the growth of unwanted pathogens as most microorganisms cannot thrive under highly acidic conditions. The work of Okereke *et al*. (2015) showed that the extracts of *H. sabdariffa* revealed the presence of secondary metabolites of plants in form of phytochemicals, vitamins and vital minerals which have curative and antimicrobial properties. One of the major phytochemicals present in the calyces of *H. sabdariffa* are flavonoids which exhibits antibacterial activity by damaging the cell walls of bacteria and eventual lysing of the nucleus (Cushnie & Lamb 2005).

The spoilage organisms isolated and identified from the tomato fruit includes *Bacillus subtilis*, *Bacillus* species, *Candida krusei* and other species of *Candida.* This agrees with the work of Mbajiuka *et al.* (2014) who isolated *Bacillus subtilis, Candida* species and other spoilage microorganisms from tomato and pawpaw fruits. His study also showed that most of the spoilage organisms frequently gain access into the fruits during the process of cultivating, harvesting, grading and packaging. It is therefore important that care be taken in the handling of tomatoes from the time of cultivation to the point of sale and distribution by farmers and traders. Moreover, previous studies had revealed that biodeterioration of the tomato fruits was influenced by the cell wall degrading enzymes secreted by the spoilage microorganisms which utilise the plant cell wall as nutrient sources *(*Al-Hindi *et al.* 2011).

## CONCLUSION

The result obtained from this work has shown that the plant extracts of *A. muricata* and *H. sabdariffa* are able to extend the shelf life and also retain the quality of tomato fruits. This study established the potency of the aqueous extracts of *A. muricata* which proved effective preserving up to 50% of the tomatoes 35 days after treatment. *A. muricata* and *H. sabdariffa* are known to contain several phytochemicals that helped in the preservation of the tomato fruits. Therefore, this study has established the use of plant extracts in extending the shelf life of perishable tomato fruits thus minimising wastes and economic loss to the farmers and country in general.

## Figures and Tables

**Table 1:** The effects of *A. muricata* leaf, bark and seed powder on preservation of tomato fruits.

Time in days	Leaf	Bark	Seed	Control	*p*-value
5	100	100	8 (100)	8 (100)	0.563
10	100	87.5	7 (87.5)	3 (37.5)	0.032
5	75	62.5	6 (75)	1 (12.5)	0.002
20	75	37.5	5 (62.5)	0 (0.0)	0.002
25	62.5	37.5	3 (37.5)	0 (0.0)	0.003
30	50	0.0	3 (37.5)	0 (0.0)	0.001
35	50	0.0	0 (0.0)	0 (0.0)	0.002
Mean preservation rate	73.21	46.43	57.14	21.43	0.021

*Note*: *p* < 0.05

**Table 2:** The effects of ethanolic extract of leaf, bark and seeds *A. muricata* on preservation of tomato fruits.

Time in days	Leaf	Bark	Seed	Control	*p*-value
5	8 (100)	8 (100)	8 (100)	8 (100)	0.645
10	6 (75)	6 (75)	6 (75)	3 (37.5)	0.024
15	4 (50)	5 (62.5)	5 (62.5)	1 (12.5)	0.001
20	3 (37.5)	4 (50)	4 (50)	0 (0.0)	0.001
25	3 (37.5)	4 (50)	3 (37.5)	0 (0.0)	0.002
30	2 (25)	3 (37.5)	2 (25)	0 (0.0)	0.001
35	0 (0.0)	0 (0.0)	2 (25)	0 (0.0)	0.003
Mean preservation rate	46.43	57.14	53.57	21.43	0.001

*Note*: *p* < 0.05

**Table 3:** The effects of aqueous extract of the leaf, bark and seed of *A. muricata* and the calyces of *H. sabdariffa* on the preservation of tomato fruits.

Time in days	Leaf	Bark	Seed	Roselle plant	Control	*p*-value
5	8 (100)	8 (100)	8 (100)	8 (100)	8 (100)	NA
10	7 (87.5)	7 (87.5)	8 (100)	8 (100)	3 (37.5)	0.002
15	6 (75)	6 (75)	6 (75)	6 (75)	1 (12.5)	0.072
20	5 (62.5)	5 (62.5)	5 (62.5)	6 (75)	0	0.026
25	4 (50)	4 (50)	3 (37.5)	4 (50)	0	0.040
30	3 (37.5)	3 (37.5)	3 (37.5)	4 (50)	0	0.052
35	3 (37.5)	2 (25)	2 (25)	4 (50)	0	0.001
Mean preservation rate	64.30	62.50	62.50	71.43	21.43	0.001

*Note*: *p* < 0.05

**Table 4:** The effects of different concentrations of the aqueous extracts of *A. muricata* leaves on preservation of tomato fruits.

Time in days	3 g	5 g	6 g	9 g	7 g	*p*-value
2	4(100)	4(100)	4(100)	4(100)	4(100)	NA
4	4(100)	3(75)	4 (100)	3(75)	4(100)	0.064
7	4(100)	2(50)	4(100)	3(75)	3(75)	0.026
9	3(75)	2(50)	4(100)	2(50)	3(75)	0.001
12	3(75)	1(25)	3(75)	2(50)	2(50)	0.003
15	2(50)	0(0.0)	3(75)	1(25)	2(50)	0.002
Mean preservation rate	83.33	50	91.70	62.5	75	0.001

*Note*: *p* < 0.05

**Table 5:** Comparative studies of the mean preservation rates of tomato fruits with different treatments of *A. muricata*.

	Powder	Ethanol	Aqueous	*p*-value
Leaf	73.21	46.43	64.3	0.002
Bark	46.43	57.14	62.5	0.021
Seed	57.14	53.57	62.5	0.548
Control	21.43	21.43	21.43	nd

*Note*: *p* < 0.05; nd = not determined.

**Table 6:** Identification of spoilage yeasts from tomato fruits.

SN	Macroscopy	Microscopy	GGT	Urea	Cy-Hex	Growth @37	Glu	Yeast
Plate 7	Creamy colour, smooth and glabrous	Small, elongated ovoid budding blastoconidia cells	−	−	−	+	+	*Candida krusei*
Plate 5	White chalky, slightly fluffy, rough and glabrous	Slight large, elongated ovoid budding blastoconidia cells	−	−	−	+	+	*Candida* sp.
Plate 2	Creamy colour, smooth and glabrous	Small, elongated ovoid budding blastoconidia cells	−	−	−	+	+	*Candida krusei*
Plate 4	Creamy colour, smooth and glabrous	Large, slightly elongated ovoid budding blastoconidia cells	−	−	−	+	+	*Candida* sp.

*Notes*: GGT= Germ Tube Test, Urea = Urea Test, Cyhex = Cycloheximide, Glu = Glucose, + = Positive reaction, − = Negative reaction

**Table 7:** Identification of spoilage bacteria from tomato fruits.

LABEL	Gram	Motility	Glucose	Lactose	Mannitol	Maltose	Indole	Methyl Red	Vogesproskauer	Citrate	H_2_ S	Sucrose	Urea	Oxidase	Coagulase	Catalase	ISOLATE
PL 6	GPB	+	+	+	+	+	−	−	+	−	−	+	−	−	NA	+	*Bacillus subtilis*
PL 3	GPB	+	+	+	+	+	−	−	+	−	−	+	−	−	NA	+	*Bacillus* sp.
PL 1	GPB	+	+	+	+	+	−	−	+	−	−	+	−	−	NA	+	*Bacillus* sp.

*Notes*: + = Positive reaction, − = Negative reaction, N A= Not applicable, NG = No growth, PL = Plate

## References

[b1-tlsr-33-1-1] Abdul-Wahab SM, Jantan I, Haque MA, Arshad L (2018). Exploring the leaves of *Annona muricata* L. as a source of potential anti-inflammatory and anticancer agents. Frontiers in Pharmacology.

[b2-tlsr-33-1-1] Adefegha SA, Oyeleye SI, Oboh G (2015). Distribution of phenolic contents, antidiabetic potentials, antihypertensive properties, and antioxidative effects of Soursop (*Annona muricata* L.) fruit parts *in-vitro*. Biochemistry Research International.

[b3-tlsr-33-1-1] Ajayi A (2013). Nature of tomatoes micro flora under storage. American Journal of Experimental Agriculture.

[b4-tlsr-33-1-1] Al-Hindi RR, Al-Najada AR, Mohamed SA (2011). Isolation and identification of some fruit spoilage fungi: Screening of plant cell wall degrading enzymes. African Journal of Microbiology Research.

[b5-tlsr-33-1-1] Al-Wandawi H, Al-Shaikhaly K, Abdu-Rahman M (1984). Roselle seeds: A new source of protein. Journal of Agricultural and Food Chemistry.

[b6-tlsr-33-1-1] Babalola SO, Babalola AO, Aworh OC (2001). Compositional attributes of the calyces of Roselle (*Hibiscus sabdariffa L*.). Journal of Food Technology in Africa.

[b7-tlsr-33-1-1] Baiyewu RA, Amusa NA, Ayoola OA, Babalola OO (2007). Survey of the postharvest diseases and aflatoxin contamination of marketed pawpaw fruit (*Carica papaya* L) in South Western Nigeria. African Journal of Agricultural Research.

[b8-tlsr-33-1-1] Balami YA (1998). The effect of processing conditions packaging and storage on selected quality attributes of “MungzaNtusa”. PhD diss.

[b9-tlsr-33-1-1] Bautista-Banos S, Barrera-Necha LL, Bravo-Luna L, Bermudez-Torres K (2002). Antifungal activity of leaf and stem extracts from various plant species on the incidence of *Colletotrichum gloeosporioides* of papaya and mango fruit after storage. Mexican Journal of Phytopathology.

[b10-tlsr-33-1-1] Borras II, Fernandez AS, Arraez RD, Palmeros SPA, Del Val DR, Andrade GI, Segura CA (2015). Characterization of phenolic compounds, anthocyanidins, antioxidant and antimicrobial activity of 25 varieties of Mexican Roselle (*Hibiscus sabdariffa*). Industrial Crops and Products.

[b11-tlsr-33-1-1] Brunold C, Deters A, Knoepfel-Sidler F, Hafner JM, Hensel A (2004). Polysaccharides from *Hibiscus sabdariffa* flowers stimulate proliferation of human Keratinocytes plants. Medicinal plants.

[b12-tlsr-33-1-1] Chessbrough M (1991). Mycology, medical laboratory manual for tropical countries.

[b13-tlsr-33-1-1] Chessbrough M (1994). Medical laboratory manual for tropical countries. Vol. II microbiology.

[b14-tlsr-33-1-1] Cowan ST, Steel KJ, Barrow GI, Felthan RKA (1993). Enterobacteriacea. Manual for the identification of bacteria.

[b15-tlsr-33-1-1] Cushnie TP, Lamb AJ (2005). Antimicrobial activity of flavonoids. International Journal of Antimicrobial Agents.

[b16-tlsr-33-1-1] Ebimieowei E, Nwauzoma A, Bawo D (2013). Post-harvest spoilage of tomatoes (*Lycopersicum esculentum*) and control strategies in Nigeria. Journal of Biology, Agriculture and Healthcare.

[b17-tlsr-33-1-1] Edeoga HO, Okwu DE, Mbaebie BO (2005). Phytochemical constituents of some Nigerian medicinal plants. African Journal of Biotechnology.

[b18-tlsr-33-1-1] Ejale A, Abdullah H (2004). Preservation of ripe tomato (*Lycopersicum esculentum Mill*) fruits with dried leaf powder of Neem (*Azadirachta indica A. Juss*). Nigerian Journal of Applied Science.

[b19-tlsr-33-1-1] Gajalakshmi S, Vijayalakshmi S, Rajeswari D (2012). Phytochemical and pharmacological Properties of *Annona muricata*: A review. International Journal of Pharmacy and Pharmaceutical Sciences.

[b20-tlsr-33-1-1] Garg RK, Batav N, Silawat N, Singh RK (2013). Isolation and identification of pathogenic microbes from tomato puree and their delineation of distinctness by molecular techniques. Journal of Applied Biology and Biotechnology.

[b21-tlsr-33-1-1] Gavamukulya Y, Abou-Elella F, Wamunyokoli F, Ael-Shemy H (2014). Phytochemical screening, anti-oxidant activity and *in vitro* anticancer potential of ethanolic and water leaves extracts of *Annona muricata* (Graviola). Asian Pacific Journal of Tropical Medicine.

[b22-tlsr-33-1-1] Hosea ZY, Liamngee K, Owoicho AL, Agatsa TD (2017). Effect of Neem leaf powder on post-harvest shelf life and quality of tomato fruits in storage. International Journal of Development and Sustainability.

[b23-tlsr-33-1-1] Ijato J, Otoide J, Ijadunola J, Aladejimokun A (2011). Efficacy of antimicrobial effect of *Vernonia amygdalina* and *Tridax procumbens* in *in-vitro* control of tomato (*Lycopersicon esculentum*) post-harvest fruit rot. Report and Opinion.

[b24-tlsr-33-1-1] Irokanulo E, Egbezein I, Owa S (2015). Use of *Moringa oleifera* in the preservation of fresh tomatoes. IOSR Journal of Agricultural and Veterinary Science.

[b25-tlsr-33-1-1] Jannah R, Husni MA, Nursanty R (2017). Inhibition test of methanol extract from soursop leaf (*Annona muricata* Linn.) against *Streptococcus mutans* bacteria. Jurnal Natural.

[b26-tlsr-33-1-1] Kator L, Oche OD, Hosea ZY, Agatsa TD (2019). Effect of aqueous extract of moringa leaves on postharvest shelf life and quality of tomato fruits inoculated with fungal pathogens in Makurdi. Asian Journal of Agricultural and Horticultural Research.

[b27-tlsr-33-1-1] Maddox DA (1998). Implications of new technologies for seed health testing and the worldwide movement of seed. Seed Science Research.

[b28-tlsr-33-1-1] Mady C, Manuel D, Mama S, Augustin N, Max R, Oumar S (2009). The bissap (*Hibiscus sabdariffa*): Composition and principal uses. Fruits.

[b29-tlsr-33-1-1] Maseret D, Ali M, Kassahun B (2012). Evaluation of tomato (*Lycopersicum esculentum Mill*) genotypes for fruit quality and shelf life. The African Journal of Plant Science and Biotechnology.

[b30-tlsr-33-1-1] Mbajiuka S, Emmanuel C, Emmanuel E (2014). Isolation of microorganisms associated with deterioration of tomato (*Lycopersicum esculentum*) and pawpaw (*Carica papaya*) fruits. International Journal of Current Microbiology and Applied Sciences.

[b31-tlsr-33-1-1] Mishra M, Shukla YN, Jain SP, Kumar S (1999). Chemistry and pharmacology of some *Hibiscus* species. Journal of Medicinal and Aromatic Plant Sciences.

[b32-tlsr-33-1-1] Ogundipe F, Bamidele F, Adebayo-Oyetoro A, Ogundipe O, Tajudeen O (2012). Incidence of bacteria with potential public health implications in raw *Lycopersicon esculentum* (tomato) sold in Lagos State, Nigeria. Nigerian Food Journal.

[b33-tlsr-33-1-1] Okereke CN, Iroka FC, Chukwuma MO (2015). Phytochemical analysis and medicinal uses of *Hibiscus Sabdariffa*. International Journal of Herbal Medicine.

[b34-tlsr-33-1-1] Olaleye MT (2007). Cytotoxicity and antibacterial activity of methanolic extract of *Hibiscus sabdariffa*. Journal of Medicinal Plants Research.

[b35-tlsr-33-1-1] Pai BH, Rajesh G, Shenoy R, Rao A (2016). Anti-microbial efficacy of soursop leaf extract (*Annona muricata*) on oral pathogens: An *in-vitro* study. Journal of Clinical and Diagnostic Research.

[b36-tlsr-33-1-1] Panthak P, Saraswathy D, Vora A, Saval J (2010). In vitro antimicrobial activity and phytochemical analysis of the leaves of *Annona muricata*. International Journal of Pharmacy Research and Development.

[b37-tlsr-33-1-1] Ploey A, Heuvelink E (2005). Influence of sub-optimal temperature and tomato growth and yield: A review. Journal of Horticultural Science and Biotechnology.

[b38-tlsr-33-1-1] Pomper KW, Lowe JD, Crabtree SB, Keller W (2009). Identification of annonaceous acetogenins in the ripe fruit of the North American pawpaw (*Asimina triloba*). Journal of Agricultural and Food Chemistry.

[b39-tlsr-33-1-1] Raheja S, Thakore B (2002). Effect of physical factor, plant extracts and bio agent on *Colletotrichum gloeosporioides* Penz, the causal organism of anthracnose of Yam. Journal of Mycology and Plant Pathology.

[b40-tlsr-33-1-1] Rahman FA, Haniastuti T, Utami TW (2017). The effect of ethanol extract of soursop leaf (*Annona muricata* L.) on Adhesion of Streptococcus mutans ATCC 35668 to hydroxyapatite discs. Majadih Kedokteran Gigi Indonesia.

[b41-tlsr-33-1-1] Raybaudi R, Alirica I, Francisco A, Felipe S, Jonathan M, Alenxandra Z, Maria I (2015). An analysis In-vitro of the cytotoxic, antioxidant and antimicrobial activity of aqueous and alcoholic extracts of *Annona muricata Linn* seed and Pulp. British Journal of Applied Sciences and Technology.

[b42-tlsr-33-1-1] Rosemary I, Uchegbu R, Kalu U, Irenus C, Jacinta N (2017). Evaluation of the antimicrobial activity and chemical composition of the leaf extract of *Annona muricata Linn* (Soursop) grown in Eastern Nigeria. Archives of Current Research International.

[b43-tlsr-33-1-1] Sari DP, Basyuni M, Hasibuan PA, Wati R (2018). The inhibition of polyisoprenoids from *Nypa fruticans* leaves on cyclooxygenase 2 expression of widr colon cancer cells. Asian Journal of Pharmaceutical and Clinical Research.

[b44-tlsr-33-1-1] Singh H, Korpraditskul D, Singh P (1999). Evaluation of some plant extracts for control of Colletotrichum capsici the causal agent of *Chilli anthracnose.*. Journal of Science and Food Agriculture.

[b45-tlsr-33-1-1] Sood M, Kaul R, Bhat A, Singh A, Singh J (2011). Effect of harvesting methods and postharvest treatments on quality of tomato. Journal of Food Science and Technology.

[b46-tlsr-33-1-1] Thirupathi V, Sasikala S, John K (2006). Preservation of fruits and vegetables by wax coating. Journal of Science and Food Agriculture.

[b47-tlsr-33-1-1] Viera GH, Mourao JA, Angelo AM, Costa RA (2010). Antibacterial effect (in-vitro) of *Moringa oleifera* and *Annona muricata* against Gram positive and Gram negative bacteria. Journal of the São Paulo Institute of Tropical Medicine.

[b48-tlsr-33-1-1] Vijayameena C, Subhashini G, Loganayagi M, Ramesh B (2013). Phytochemical screening and assessment of antibacterial activity for the bioactive compounds of *Annona muricata.*. International Journal Current Microbial Applied Sciences Research.

[b49-tlsr-33-1-1] World Health Organization (1983). Laboratory biosafety manual.

[b50-tlsr-33-1-1] Wogu MD, Ofuase O (2014). Microorganisms responsible for the spoilage of tomato fruits, *Lycopersicum esculentum*, sold in markets in Benin City, Southern Nigeria. Scholars Academic Journal of Biosciences.

[b51-tlsr-33-1-1] Wong PK, Yusof S, Ghazali HM, Man YBC (2002). Physico-chemical characteristics of Roselle (*Hibiscus sabdariffa* L.). Journal of Nutrition and Food Sciences.

[b52-tlsr-33-1-1] Yeboah AK (2011). A survey on postharvest handling, preservation and processing methods of tomato (*Solanum lycopersicum*) in the Dormaa and Tano South Districts of the Brong Ahafo Region of Ghana. PhD diss.

[b53-tlsr-33-1-1] Yin MC, Chao CY (2008). Anti-campylobacter, anti-aerobic, and anti-oxidative effects of Roselle Calyx extract and protocatechuic acid in ground beef. International Journal of Food Microbiology.

[b54-tlsr-33-1-1] Zafra-Polo CM, Figadère B, Gallardo T, Tormo J, Cortes D (1998). Natural acetogenins from annonaceae, synthesis and mechanisms of action. Photochemistry.

